# Antibacterial, antidiarrhoeal, and cytotoxic activities of methanol extract and its fractions of *Caesalpinia bonducella* (L.) Roxb leaves

**DOI:** 10.1186/1472-6882-13-101

**Published:** 2013-05-12

**Authors:** Muhammad Mutassim Billah, Rafikul Islam, Hajera Khatun, Shahnaj Parvin, Ekramul Islam, SM Anisul Islam, Akbar Ali Mia

**Affiliations:** 1 Department of Pharmacy, International Islamic University Chittagong, Chittagong 4203, Bangladesh; 2 Department of Pharmacy, South East University, Dhaka 1213, Bangladesh; 3 Department of Pharmacy, University of Rajshahi, Rajshahi 6205, Bangladesh

**Keywords:** *Caesalpinia bonducella*, Antimicrobial, Antidiarrhoeal, Cytotoxicity

## Abstract

**Background:**

*Caesalpinia bonducella* is an important medicinal plant for its traditional uses against different types of diseases. Therefore, the present study investigated the antimicrobial, antidiarrhoeal, and cytotoxic activities of the methanol extract and ethyl acetate, chloroform, and petroleum ether (pet. ether) fractions of *C. bonducella* leaves.

**Methods:**

The antibacterial potentialities of methanol extract and its fractions of *C. bonducella* leaves were investigated by the disc diffusion method against four gram-positive and five gram-negative bacteria at 300, 500 and 800 μg/disc. Kanamycin (30 μg/disc) was used as the standard drug. Antidiarrhoeal activities of leaf extracts were evaluated at two doses (200 and 400 mg/kg) and compared with loperamide in a castor oil-induced diarrhoeal model in rat. The fractions were subjected to a brine shrimp lethality test to evaluate their cytotoxicity.

**Results:**

The methanol extract and other three fractions exhibited better activities at higher concentrations. Amongst, the chloroform fraction showed maximum activity at all three concentrations (300, 500, and 800 μg/disc) against almost all bacteria. *S. aureus* and *P. aeruginosa* showed better sensitivities to all extracts at all three concentrations excluding the pet. ether fraction. *Bacillus megaterium* and *Klebsiella* spp. were two bacteria amongst nine that showed lowest sensitivity to the extracts. Maximum zone of inhibition (25-mm) was obtained by the methanol extract at an 800 μg/disc concentration against *S. aureus*. In the antidiarrhoeal test, all fractions exhibited dose-dependent actions, which were statistically significant (p < 0.05). Ethyl acetate fraction exerted maximum inhibition (51.11%) against defecation, whereas 57.75% inhibition was obtained for loperamide. Moderate cytotoxicity was found for the methanol extract and its three fractions compared with the standard drug vincristine sulfate in the brine shrimp bioassay. In the present study, the LC_50_ values of the methanol crude extract and ethyl acetate, chloroform, pet. ether fractions and vincristine sulfate were 223.87, 281.84, 112.2, 199.53, and 12.59 μg/mL, respectively. Therefore, the ethyl acetate fraction showed maximum cytotoxicity, whereas minimum cytotoxicity was observed for the chloroform fraction.

**Conclusion:**

The present study revealed that the ethyl acetate fraction of the *C. bonducella* leaves has significant antidiarrhoeal properties. The methanol extract and other three fractions of the *C. bonducella* leaves possess potent antibacterial activities along with moderate cytotoxicities that may lead to new drug development.

## Background

*Caesalpinia bonducella* L. (Family: Fabaceae) is an important medicinal plant, which is widely distributed in the tropical and subtropical regions of Asia and the Caribbean [1]. In Bangladesh, this plant is abundant in forests and village thickets of Dhaka, Chittagong, Khulna, Tangail, and North Bengal. The plant is known as Nata in Bengali and fever nut or nicker nut in English. Different parts of the plant have extensive uses in folk medicines for the treatment of a variety of diseases. It has been reported that seeds of the plant possess antidiarrhoeal, antiviral, antibacterial, antimicrobial, antifungal, antidiabetic, antitumor, antipyretic and analgesic, antifilarial, anxiolytic, anti-inflammatory, antioxidant, immunomodulatory, adaptogenic, anticonvulsant, antispasmodic, nootropic, antifeedant, antiamoebic, antioestrogenic, diuretic, insecticidal, as well as trypsin and chymotrypsin inhibitor properties [2-8]. Phytochemical analysis of *C. bonducella* seeds has revealed the presence of alkaloids, flavonoids, glycosides, saponins, tannins, and triterpenoids. *C. bonducella* fruits has been found to have significant antidiarrhoeal activity in mice [9]. Devi *et al.* reported that the flowers of *C. bonducella* possess analgesic and antipyretic properties [10]. Phytochemical studies on ethanolic extracts of the bark of *C. bonducella* yielded two new homoisoflavonoids along with five known natural products. All of these compounds exhibited different levels of glutathione S-transferase (GST) inhibitory and antifungal potentials [11]. Antinociceptive, antidiarrhoeal, and CNS depressant activities of the ethanolic extract of *C. bonducella* stem were also observed by Ahmed *et al*. [12]. Recently, it has been studied that plant root extract possesses anti-inflammatory and antimicrobial activities, whereas root bark shows antifertility effects [13,14]. Leaves of the plant exhibit anthelmintic, anti-inflammatory, analgesic, antipyretic, CNS depressant antiproliferative, antipsoriatic, anti-amyloidogenic, hepatoprotective, antioxidant, antitumor, mosquito larvicidal, antiasthmatic, and muscle contractile properties [15-27]. Ahsan *et al*. found antibacterial and cytotoxic activities of the methanolic leaf and bark extracts of the plant [28]. A polymethylene compound, which is responsible for antimicrobial activity, was isolated from an ethyl acetate extract of *C. bonducella* leaves [29]. Antidiarrhoeal activity of the ethanolic extract of leaves of the plant was evaluated by Ahmed *et al*. [12]. Toxicity was found at higher doses but not at low doses of the leaf extracts in different animal model experiments [30-32]. There are some reports of antibacterial, antidiarrhoeal, and cytotoxic activities of *C. bonducella* leaves. To date, different fractions of leaf extracts have not been systemically studied. Therefore, the present investigation was performed.

## Methods

### Plant material

For this present investigation, *C. bonducella* leaves were collected from Mirpur, Dhaka, Bangladesh in October, 2010 and identified by experts of the Bangladesh National Herbarium, Dhaka, where a voucher specimen has also been retained with accession no. 3803. The collected plant parts were dried for one week and pulverized into a coarse powder using a suitable grinder. The powder was stored in an airtight container and kept in a cool, dark, and dry place until further analysis.

### Extract preparation

Approximately 750 g of powdered material was placed in a clean, flat-bottomed glass container and soaked in methanol. The container with its contents was sealed and kept for 7 days accompanied by occasional shaking and stirring. The entire mixture then underwent a coarse filtration by a piece of clean, white cotton material. The extract then was filtered through Whatman filter paper (Bibby RE200, Sterilin Ltd., UK) and was concentrated to obtain the methanol crude extract (9.5 g), which was divided into two portions. One portion (1.5 g) was poured into glass vials to be tested as crude methanol extract, whereas the second portion (8 g) was dissolved in 100 mL methanol and partitioned successively with ethyl acetate, chloroform, and pet. ether. The fractions were then concentrated using a rotary evaporator to give ethyl acetate fraction (yield weight 2.50 g), chloroform fraction (yield weight 1.25 g), and pet. ether fraction (yield weight 2.15 g). This process rendered a gummy concentrated reddish black colour. The gummy extracts were transferred to a closed container for further use and storage.

### Animals

Young Long-Evans rats of either sex weighing approximately 80-120 g were used for this experiment. The rats were purchased from the animal Research Branch of the International Centre for Diarrhoeal Disease and Research, Bangladesh (ICDDRB). After their purchase, the rats were kept in standard environmental conditions (24.0 ± 0°C & 55-65% relative humidity and 12 h light/dark cycle) for one week to acclimate and fed ICDDRB formulated rodent food and water *ad libitum*. The experimental procedures involving animals were conducted in accordance with the guidelines of Institute of Biological Sciences, University of Rajshahi, Bangladesh. The study protocol was approved by Institutional Animal, Medical Ethics, Biosafety and Biosecurity Committee (IAMEBBC) at the Institute of Biological Sciences, University of Rajshahi, Bangladesh.

### Antibacterial assay

The methanol crude extract and the ethyl acetate, chloroform, and pet. ether fractions of the plant were screened at three concentrations (300, 500, and 800 μg/disc) against four gram-positive and five gram-negative bacteria (Table 1) using the disc diffusion method [33]. Solutions of known concentration (10 mg/mL) of the test samples were prepared by dissolving measured amounts of samples in calculated solvent volumes. Dried and sterilized filter paper discs (6-mm diameter) were then impregnated with known amounts of the test substances using a micropipette. Discs containing the test material were placed on nutrient agar medium (Merck) uniformly seeded with the pathogenic test microorganisms. The prepared inoculum size was approximately 10^6^ cfu/mL. Standard antibiotic discs (kanamycin, 30 μg/disc) and blank discs (impregnated with solvents) were used as positive and negative controls, respectively. These plates were then, kept at 4°C for a 1-h diffusion of the test material. There was a gradual change in concentration surrounding the discs. The plates were then, incubated at 37°C for 24 h to allow organism growth. The test materials having antibacterial activity inhibited microorganism growth, and a clear, distinct zone of inhibition surrounding the discs was visualized. The antibacterial activity of the test agents was determined by measuring the diameter of the zone of inhibition expressed in millimetres.

**Table 1:** Tested microbes

**Gram-negative**	**Gram-positive**
*Salmonella typhi*	*Staphylococcus aureus*
*Shigella dysenteriae*	*Bacillus subtilis*
*Pseudomonas aeruginosa*	*Bacillus cereus*
*Escherichia coli*	*Bacillus megaterium*
*Klebsiella* spp.	

### Castor oil-induced diarrhoeal test

The antidiarrhoeal effects of plant extracts were performed according to the method described by Shoba and Thomas [34]. Rats fasted for 24 h and were divided into ten groups (n = 4). Group I received 10 mL/kg of Tween 80 (1% Tween 80 in water) orally and served as control animals. Animals in group II received loperamide (5 mg/kg, p.o.) and served as the standard treatment group, whereas groups III, IV, V, VI, VII VIII, IX, and X received orally 200 or 400 mg/kg of methanol, ethyl acetate, chloroform, or pet. ether extract, respectively. The extracts were suspended in Tween 80 (1% v/v). One hour after oral administration of treatments, the animals received castor oil (1 mL) orally, and they were individually placed in cages, the floor of which lined with blotting paper for observation of the number and consistency of faecal droppings. The numbers of both wet and dry droppings were counted every 60 min for 4 h, and the white paper was changed after each evaluation. The means of the stools passed by the treated groups were compared with that of the control group. The mean number of diarrhoeic faeces pooled by the control group was considered as 100%. The level of inhibition (%) of defecation caused by extracts was calculated relative to the control using the following relationship: Inhibition of defecation (%) = [(NDC - NDT)/NDC] × 100; where NDC = mean number of diarrhoeic faeces of the control group; NDT = mean number of diarrhoeic faeces of the treated group.

### Cytotoxicity screening

Cytotoxicity of the methanol extract and other three fractions was evaluated by the brine shrimp lethality bioassay, which is widely used for screening bioactive compounds [35,36]. In this study, a simple zoological organism (*Artemia salina*) was used as a convenient monitor for the screening. The eggs of the brine shrimp were collected from an aquarium shop (Dhaka, Bangladesh) and hatched in artificial seawater (3.8% NaCl solution) for 48 h to develop into larval srimp called nauplii. The cytotoxicity assay was performed on the brine shrimp nauplii using the Meyer method. The test samples (extract) were prepared by dissolving them in DMSO (not more than 50 μL in 5 mL solution) plus seawater (3.8% NaCl in water) to attain concentrations of 10, 50, 100, 150, 200 and 300 μg/mL^-1^. A vial containing 50 μL DMSO diluted to 5 mL was used as a control. Standard vincristine sulfate was used as a positive control. Mature shrimps were placed into each of the experimental vials. After 24 h, the vials were inspected using a magnifying glass, and the number of surviving nauplii in each vial was counted. From these data, the percent (%) of lethality of the brine shrimp nauplii was calculated for each concentration using the following formula:

$$\%\;\mathrm{Mortality}\;=\;\frac{N_{t}}{N_{0}}\;\times \;100$$

Where N_t_ = Number of dead nauplii after a 24-h incubation; N_0_ = Number of total nauplii transferred i.e., 10.

The LC_50_ (median lethal concentration) was determined from the log concentration versus % mortality curve.

## Results

### Antibacterial assay

The antibacterial activities of the methanol extract and ethyl acetate, chloroform, and pet. ether fractions of the *C. bonducella* leaves obtained by the disc diffusion method are presented in Table 2. The extracts showed different zones of inhibition at three different concentrations (300, 500, and 800 μg/disc) against four gram-positive and five gram-negative bacteria. All extracts exerted better activities at an 800 μg/disc concentration against the tested microorganisms. However, at 300 μg/disc, the extracts except chloroform showed no significant sensitivity against almost all of the microorganisms. The chloroform fraction exhibited better antimicrobial activity at all three concentrations (300, 500, and 800 μg/disc) against almost all bacteria. The methanol extract exerted the lowest antibacterial activity followed by the ethyl acetate and pet. ether fractions. *S. aureus* and *P. aeruginosa* exhibited better sensitivities to all extracts at all three concentrations excluding the pet. ether fractions. *B. megaterium and Klebsiella* spp. were two bacteria amongst nine that had the lowest sensitivity to the plant extracts. The maximum zone of inhibition was obtained for the methanol extract at all three concentrations against *S. aureus*.

**Table 2:** **Antibacterial effects of different *C. bonducella *leaf extract fractions**

**Name of the bacteria**	**Zone of inhibition (mm)**												
	**Kanamycin disc (30 mg/disc)**	**Methanol (μg/disc)**			**Ethyl acetate (μg/disc)**			**Chloroform (μg/disc)**			**Pet. ether (μg/disc)**		
		**300**	**500**	**800**	**300**	**500**	**800**	**300**	**500**	**800**	**300**	**500**	**800**
**Gram-positive**													
*S. aureus*	36	16	21	25	-	6	6.5	7.5	12	16	-	-	6.5
*B. subtilis*	26	-	-	-	-	-	8	6.5	8	9.5	-	-	-
*B. cereus*	29	-	-	-	6.5	8	10	7	9	10	-	-	6.5
*B. megaterium*	35	-	-	-	-	-	-	-	7	8	-	-	6.5
**Gram-negative**													
*S. typhi*	29	-	-	6.5	-	-	7	7	10	13	-	6.5	7
*S. dysenteriae*	35	-	-	-	-	7	8	6.5	7.5	9	-	-	-
*P. aeruginosa*	30	6.5	6.8	7	6.5	8	8.5	6.5	7	7.5	-	-	-
*E. coli*	32	-	-	-	-	-	-	6.5	7	8	7	7.5	8
*Klebsiella* spp.	35	-	-	-	-	-	-	-	7	8	-	-	6.5

-: no activity.

### Castor oil-induced diarrhoeal test

Data are presented as the number of defecation and percent inhibition compared with the control group in Table 3. After a 30-min administration of castor oil, diarrhoea was clinically apparent for the next 4 h in the control group. This condition was markedly reduced by 57.78% by loperamide at a dose of 5 mg/kg. All of our extracts also demonstrated statistically significant (P < 0.05) inhibition of castor oil-induced diarrhoea in a dose-dependent manner. Amongst four extracts, the ethyl acetate fraction had better activity against diarrhoea and produced 51.11% inhibition at 400 mg/kg, which was approximately the percent inhibition of the standard drug (57.78%).

**Table 3:** Antidiarrhoeal activity of methanolic extract and fractions against castor oil-induced diarrhoea in rats

**Group**	**Dose (p.o.)**	**No. of faeces in 4 h**	**% inhibition of diarrhoea**
Control	10 mL/kg, p.o.	22.5 ± 2.4	-
Loperamide	5 mg/kg, p.o.	9.5 ± 0.64*	57.78
Methanol	200 mg/kg, p.o.	15 ± 1.1*	33.33
	400 mg/kg, p.o.	11.5 ± 0.09*	48.89
Ethyl acetate	200 mg/kg, p.o.	16.25 ± 1.38*	27.78
	400 mg/kg, p.o.	11 ± 1.58*	51.11
Chloroform	200 mg/kg, p.o.	12 ± 1.29*	46.67
	400 mg/kg, p.o.	11.25 ± 1.25*	50
Pet. ether	200 mg/kg, p.o.	15.5 ± 1.76*	31.11
	400 mg/kg, p.o.	13.75 ± 1.38*	38.89

All values are expressed as mean ± SEM (n = 5); One-way analysis of variance (ANOVA) followed by Dunnett’s test. *P < 0.05, significant compared with the control samples.

### Brine shrimp lethality bioassay

**Figure 1:**
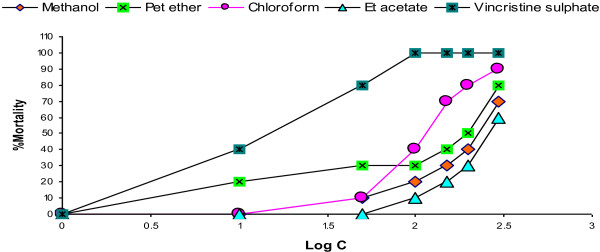
**Brine shrimp lethality bioassay.** Determination of LC_50_ values for methanol extract and chloroform, ethyl acetate and pet. ether fractions of *C. bonducella* leaves from linear correlation between log concentrations versus % mortality.

Following the procedure of Meyer, the lethality of the methanol crude extract and its three fractions of *C. bonducella* leaves were determined on *Artemia salina* after sample exposure for 24 h. The negative control (vehicle only) and vincristine sulfate (positive control) were also used to compare the toxic activities of the extracts. This technique was applied to determine the general toxicity of the plant extract. Percent mortality of brine shrimp at six different concentrations (10 to 300 μg/mL) of the extracts has been presented in Table 4. From Figure 1, it is clear that the % mortality is directly proportional to the extract concentrations. LC_50_ values of the methanol extract and ethyl acetate, chloroform, and pet. ether fractions obtained in the present experiment were 223.87, 281.84, 112.2, and 199.53 μg/mL, respectively. Therefore, the chloroform fractions demonstrated greater toxicity compared with others, and the ethyl acetate fractions exerted the lowest % mortality. The LC_50_ value for the standard drug vincristine sulfate was 12.59 μg/mL. However, no mortality was obtained for the negative control group.

**Table 4:** % of mortality of different fractions of the extract at six concentrations

**Concentration (μg/mL)**	**Log C**	**% mortality**				
		**Methanol**	**Ethyl acetate**	**Chloroform**	**Pet. ether**	**Vincristine sulfate**
0	0	0	0	0	0	0
10	1	0	0	0	20	40
50	1.7	10	0	10	30	80
100	2	20	10	40	30	100
150	2.18	30	20	70	40	100
200	2.30	40	30	80	50	100
300	2.47	70	60	90	80	100
LC_50_ (μg/mL)		223.87	281.84	112.2	199.53	12.59

## Discussions

The discovery of effective antibiotics, vaccines and other products or methods has decreased the devastating impact of infectious diseases and improved quality of life. However, the efficacy of many antibiotics is being threatened by the emergence of microbial resistance to existing chemotherapeutic agents because of their indiscriminate and inappropriate use [37]. The use of some antibiotics is associated with side effects, including allergy, immune suppression, and hypersensitivity [38]. Many populations who live in developing countries are deprived of the advantages of modern medicine because of its high cost; hence, poor people are more vulnerable to infectious diseases. Besides these, co-infection with multiple diseases is an obstacle to infection prevention and treatment. For all these reasons, there is a pressing need to identify new, safe, and cost-effective antimicrobial agents that would help to alleviate the problems of infectious diseases. Plant-derived natural products represent an attractive source of antimicrobial agents because they are natural and affordable, especially for rural societies [39]. Acceptance of medicines from such plant origins as an alternative form of healthcare is increasing because they are serving as promising sources of novel antibiotic prototypes. Moreover, these compounds may have different mechanisms of action than conventional drugs and could be of clinical importance to improve health care [40-42]. Some of the phytochemical compounds e.g., glycoside, saponin, tannin, flavonoids, terpenoid, and alkaloids, have been reported to have antimicrobial activity [43,44].

Despite declines in death rate, diarrhoea remains a potential cause of morbidity and mortality, especially in children in developing countries. More than 80% of childhood deaths in Africa and South Asia are due to diarrhoea [45]. In developing countries, the majority of people living in rural areas almost exclusively use traditional medicines to treat many diseases including diarrhoea. Traditional medicines, such as *Punica granatum, Psidium guajava, Ocimum gratissimum, Phyllanthus emblica,* and *Aegle marmelos,* are commonly used in Bangladesh, and their use against diarrhoeas has now been scientifically established [46-50]. Therefore, medicinal plants are a promising source of antidiarrhoeal drugs [51,52]. For this reason, WHO has encouraged studies that evaluate traditional medicinal practices to treat and prevent diarrhoeal diseases [53,54]. The present study showed that the methanol, ethyl acetate, chloroform, and petroleum ether extract of leaves of *C. bonducella* at a dose of 400 mg/kg exhibited a significant inhibition of castor oil induced diarrhoea in extract dependent manner in experimental rats. After oral ingestion of castor oil, ricinoleic acid is released by lipases in the intestinal lumen. This acid causes irritation and inflammation to the intestinal mucosa resulting in the release of inflammatory mediators, such as prostaglandins, histamine, and nitric oxide which in turn stimulates gastrointestinal motility, secretions, epithelial permeability and edema of the intestinal mucosa, thereby preventing the re-absorption of Na^+^, K^+^ and water [55,56]. Tanin, alkaloids, flavonoids, saponins, sterols and/or terpenoids present in plants are responsible for antidiarroheal activity [50]. The above constituents may be present in the fractions studied, which could exert their antidiarrhoeal action. The mechanism is not determined here.

Continuous efforts to identify new and novel bioactive materials have encouraged us to evaluate the activities of leaf fractions of *C. bonducella* against an array of microorganisms, diarrhoea, and cytotoxicity. Results have revealed that the methanol crude extract and the ethyl acetate, pet. ether, and chloroform fractions of *C. bonducella* possess better antimicrobial activities against certain microorganisms at different doses, but all samples demonstrated significant inhibition of diarrhoea in rat against castor oil-induced defecation. Non-prescription use of medicinal plants is cited today as an important health problem, in particular their nephrotoxicity [55]. Therefore, if a plant extract is found to show significant antimicrobial activity, an acceptable level of toxicity must be considered. In the present investigation, moderate brine shrimp cytotoxicities were found for all extracts compared with the standard drug vincristine sulfate. However, these activities might be due to the presence of bioactive or inhibitory compounds or factors in the fractions or synergism by the existence of some compounds or factors in the fractions. Because a variety of constituents, such as saponin, tannin, polyphenols, flavonoids, and alkaloids, may be present in the fractions studied, further extensive investigations are required to determine the active antimicrobial, antidiarrhoeal, and cytotoxic properties present in the leaf extracts.

## Conclusion

The results presented in this study indicated that different fractions of crude methanol extract of *C. bonducella* leaves possess antibacterial, antidiarrhoeal, and cytotoxic activities. These results further support the traditional use of this plant in medicine.

## Competing interests

The authors declare that they have no competing interests.

## Authors’ contribution

MMB: performed the study as his project work; MRI: supervised and coordinated the project and prepared the manuscript; HK: provided assistance to perform the antidiarrhoeal investigation of the extracts; SMAI and AAM: helped in the preparation of the extracts and experiments; EI and SP: evaluated the data and corrected the manuscript for publication. All of the authors have read and approved the manuscript.

## Pre-publication history

The pre-publication history for this paper can be accessed here:

http://www.biomedcentral.com/1472-6882/13/101/prepub
